# Predictors of child and adolescent mental health treatment outcome

**DOI:** 10.1186/s12888-022-03837-y

**Published:** 2022-03-31

**Authors:** Julian Edbrooke-Childs, Anisatu Rashid, Benjamin Ritchie, Jessica Deighton

**Affiliations:** 1 Evidence Based Practice Unit, UCL and Anna Freud Centre, 4-8 Rodney Street, London, N1 9JH UK; 2 Child Outcomes Research Consortium, Anna Freud Centre, London, UK

**Keywords:** Child and adolescent, Mental health, Treatment outcome, Case-mix

## Abstract

**Background:**

To examine the predictors of treatment outcome or improvement in mental health difficulties for young people accessing child and adolescent mental health services.

**Methods:**

We conducted a secondary analysis of routinely collected data from services in England using the Mental Health Services Data Set. We conducted multilevel regressions on *N* = 5907 episodes from 14 services (*M*_*age*_ = 13.76 years, *SD*_*age*_ = 2.45, range = 8–25 years; 3540 or 59.93% female) with complete information on mental health difficulties at baseline. We conduct similar analyses on *N* = 1805 episodes from 10 services (*M*_*age*_ = 13.59 years, *SD*_*age*_ = 2.33, range = 8–24 years; 1120 or 62.05% female) also with complete information on mental health difficulties at follow up.

**Results:**

Girls had higher levels of mental health difficulties at baseline than boys (*β* = 0.28, 95% CI = 0.24–0.32). Young people with higher levels of mental health difficulties at baseline also had higher levels of deterioration in mental health difficulties at follow up (*β* = 0.72, 95% CI = 0.67–0.76), and girls had higher levels of deterioration in mental health difficulties at follow up than boys (*β* = 0.09, 95% CI = 0.03–0.16). Young people with social anxiety, panic disorder, low mood, or self-harm had higher levels of mental health difficulties at baseline and of deterioration in mental health difficulties at follow up compared to young people without these presenting problems.

**Conclusions:**

Services seeing higher proportions of young people with higher levels of mental health difficulties at baseline, social anxiety, panic disorder, low mood, or self-harm may be expected to show lower levels of improvement in mental health difficulties at follow up.

## Background

It is known that levels of mental health difficulties in children and adolescents are increasing [12]. There is some evidence showing corresponding increases in levels of mental health difficulties for young people accessing child and adolescent mental health services [3, 4]. In general populations, young women in particular are experiencing increased levels of mental health difficulties [12]. There is a need for evidence about the characteristics that predict of treatment outcome (i.e., mental health difficulties at follow up) for young people accessing child and adolescent mental health services and, correspondingly, characteristics that account for variation in levels of mental health difficulties at baseline.

There has been increased attention on treatment outcome for young people accessing child and adolescent mental health services and what types of outcomes are most important [1, 10, 11]. Change in symptoms, functioning, and goals according to self- or parent/ carer-reported measures is the predominant metric of treatment outcome in child mental health. However, there is a need to go beyond describing treatment outcome to understand which factors are associated with different treatment outcomes. Levels of service use may be one such factor and, for example, young people with psychosis, substance use, or eating disorder have been shown to be particularly more likely to have higher levels of service use compared to young people with less severe difficulties (Edbrooke-Childs J, Rashid A, Ritchie B, Deighton J. Predictors of amoutns of child and adolescent mental health service use in admiistrative data, submitted). In addition, young people referred through certain pathways, such as social care/ youth justice, were more likely to have higher levels of service use. Moreover, evidence suggests that a range of intervention, clinician, service user, service delivery, organizational, and service system characteristics may be associated with treatment outcome [17]. In terms of service user characteristics, these may include clinician, demographic, and family characteristics [15]. Nevertheless, previous evidence has been inconsistent in the extent to which these factors were associated with treatment outcome for young people accessing child and adolescent mental health services.

One of the most consistent factors is levels of mental health difficulties at baseline. Generally, studies show that higher levels of difficulties at baseline are associated with higher levels of difficulties at follow up [14]. Therefore, to provide a complete understanding of predictors of child and adolescent treatment outcome, it is important to first examine characteristics that account for variation in levels of mental health difficulties at baseline. Notwithstanding, some studies have shown that higher levels of difficulties at baseline are associated with increased likelihood of improvement [13]. Other characteristics that have, somewhat less consistently, been shown to be associated with treatment outcome include diagnosis (e.g., psychosis, conduct disorder, hyperactivity, autism), levels of functional impairment, and an older age when accessing services [3, 4, 13–15]. It is known that structural inequalities mean that young people from minoritized ethnic groups are more likely to be referred to services through routes that are less likely to be voluntary, and it is therefore important to examine if there are similar differences in treatment outcome [5].

### Aims of this study

There were two aims of the present study. The first aim was to examine characteristics in routine data from mental health services that account for variation in levels of baseline difficulties for children and adolescents. The second aim was to then examine predictors of treatment outcome for children and adolescents accessing mental health services. To address these aims, we conducted a secondary analysis of a large administrative dataset from child and adolescent mental health services in England.

## Method

### Data preparation and procedure

Data were derived from routinely collected data extracted from the ‘community activity data package’ of the Mental Health Services Data Set by NHS Digital (years 2016–17 and 2017–18). From this extract, episodes of care were constructed, referring to periods of service use consisting of at least two care contacts and less than 180 days between care contacts (excluding text message, email, or unattended), using an approach adapted from a previous study [16]. To address the first research question, episodes of care were included in the present analysis if the age at episode start was 8–25 years, the age range that the included measures can be self-reported. Additionally, episodes of care were included if the case was closed, there was at least 50 episodes per service, and there was complete data on mental health difficulties at baseline (see Measures).

This resulted in a first dataset of *N* = 5907 episodes from 14 services with 65–1667 episodes per service (*M*_*age*_ = 13.76 years, *SD*_*age*_ = 2.45, range = 8–25 years; 3540 or 59.93% female; *M*_*number of services*_ = 870, median_number of services_ *=* 769*; SD*_*number of services*_ = 554; skew_number of services_ = 0.37); please see Table 1 for descriptive statistics on all study variables. To address the second research question, episodes of care were additionally filtered based on complete data on mental health difficulties at follow up. This resulted in a second dataset of *N* = 1805 episodes from 10 services with 53–485 episodes per service (*M*_*age*_ = 13.59 years, *SD*_*age*_ = 2.33, range = 8–24 years; 1120 or 62.05% female; *M*_*number of services*_ = 483, median_number of services_ *=* 443*; SD*_*number of services*_ = 375; skew_number of services_ = 0.88). The study was approved by the University College London Research Ethics Committee (12,689/001) and the NHS Digital Data Access Request Service (DARS-NIC-140981-R5N6Z).

**Table 1:** Descriptive statistics for all study variables

	Full sample (*N =* 5907)		Follow up sample (*N =* 1805)	
	*N*	%	*N*	%
**Deprivation**				
Least	994	16.83	279	15.46
Low	993	16.81	268	14.85
Lower	1006	17.03	284	15.73
High	1171	19.82	377	20.89
Most	1743	29.51	597	33.07
**Demographics**				
Female vs. male	3540	59.93	1120	62.05
Age (M, SD)	13.76	2.45	13.59	2.33
Ethnicity				
*Asian*	117	1.98	52	2.88
Indian	26	0.44	14	0.78
Pakistani	46	0.78	19	1.05
Bangladeshi	13	0.22	8	0.44
Asian other	32	0.54	11	0.61
*Black*	94	1.59	38	2.11
Caribbean	41	0.69	16	0.89
African	32	0.54	13	0.72
Black other	21	0.36	9	0.5
*Mixed-race*	200	3.39	56	3.1
White and Black Caribbean	73	1.24	19	1.05
White and Black African	23	0.39	9	0.5
White and Asian	46	0.78	13	0.72
Mixed-race other	58	0.98	15	0.83
*Any other ethnic group*	270	4.57	87	4.82
Chinese	6	0.1	2	0.11
Other	264	4.47	85	4.71
Not reported	883	14.95	353	19.56
White British	4210	71.27	1161	64.32
*Another White background*	133	2.25	58	3.21
White Irish	14	0.24	5	0.28
White other	119	2.01	53	2.94
**Referral source**				
Primary care	2874	48.65	957	53.02
Self-referral	286	4.84	101	5.6
Education	450	7.62	152	8.42
Social care/ youth justice	207	3.5	56	3.1
Child health	242	4.1	69	3.82
Self-referral	286	4.84	101	5.6
Education	450	7.62	152	8.42
Social care/ youth justice	207	3.5	56	3.1
Child health	242	4.1	69	3.82
A&E	416	7.04	113	6.26
Mental health	264	4.47	57	3.16
Other	573	9.7	152	8.42
Not known	595	10.07	148	8.2
**Presenting difficulties**				
Social anxiety	3468	58.71	1030	57.06
Separation anxiety	2144	36.3	618	34.24
Generalised anxiety	3604	61.01	1085	60.11
OCD	1432	24.24	432	23.93
Panic	2024	34.26	580	32.13
Agoraphobia	1550	26.24	472	26.15
Phobia	931	15.76	287	15.9
Low mood	3607	61.06	1091	60.44
Repetitive behaviour	845	14.31	242	13.41
Self-harm	2318	39.24	702	38.89
Bipolar disorder	824	13.95	245	14
Psychosis	382	6	147	8
Substance use	512	8.67	128	7
ADHD	1575	27	383	21
Conduct disorder	1586	26.85	422	23.38
Risk	907	15.35	215	11.91
Toilet problems	269	4.55	65	3.6
PTSD	1243	21.04	311	17.23
Eating disorder	998	16.9	278	15.4
Attachment	1838	31.12	496	27.48
Peer relationships	3091	52.33	878	48.64
Selective mutism	173	2.93	43	2.38
Gender identity	144	2.44	46	2.55
Developmental problems	294	4.98	72	3.99
Family relationships	3004	50.85	829	45.93
Health adjustment	414	7.01	94	5.21
Self-care	556	9.41	136	7.53
Unexplained physical problems	328	5.55	91	5.04
Emerging personality disorder	1145	19.38	308	17.06
Carer management	1863	31.54	461	25.54
**Service use**				
Number of care contacts (M, SD)	13.03	24.11	15.18	25.87

*Note. A&E* Accident and Emergency, *OCD* Obsessive compulsive disorder, *ADHD* Attention-deficit-hyperactivity-disorder, *PTSD* Post-traumatic stress disorder

### Measures

#### Deprivation

Deprivation was reported using quintiles of the Income Deprivation Affecting Children Index (IDACI) based on young people’s local area of residence (Lower Layer Super Output Area).

#### Demographic characteristics

Age, gender, and ethnicity were recorded by services as part of routine data recording. Ethnicity was captured using the categories from the 2001 Census. For the main analyses, to avoid including underpowered groups, ethnicity was grouped as follows: Any other White background, any other ethnic group, Asian, Black, mixed-race, not reported, and White British.

#### Referral source

Referral source was recorded by services using 44 categories that were grouped into nine study variables for the present analysis: primary care, self-referral, education, social care/ youth justice, child health, accident and emergency department, mental health, other, and not reported.

#### Presenting difficulties

Two sources were used to identify the presence or absence of 30 non-mutually exclusive presenting difficulties. First, the 30-item clinician-reported Current View questionnaire [8] on presenting problems were used. Second, clinician-reported ICD-10 free-text diagnoses were used, which were first mapped on to the 30 Current View presenting problems, thus creating one set of harmonised 30 presenting difficulties.

#### Mental health difficulties

Baseline and follow up mental health difficulties were assessed using five subscales using four self-reported measures summarized below. To ensure conceptual and operationalisation consistency across measures, we focussed only on those assessing depression and anxiety. To accommodate the completion of different measures, measures were transformed into *z-*scores, and when multiple measures were completed, the mean *z-*score of these measures was computed. Baseline measures were completed at the initial stages of treatment and follow up measures were completed 4-6 months later or at case closure.

1. Emotional difficulties subscale of the Strengths and Difficulties Questionnaire (SDQ) [6].
2. Depression and generalized anxiety subscales of the Revised Children’s Anxiety and Depression Scale (RCADS) [2].
3. Generalised Anxiety Disorder (GAD-7) [19], which is a 7-item questionnaire assessing symptoms of generalised anxiety.
4. Patient Health Questionnaire (PHQ-9) [18], which is a 9-item questionnaire assessing symptoms of depression.

### Analytic strategy

To investigate characteristics that account for variation in levels of baseline difficulties (research question 1), two-level multilevel regressions were performed, with child as the level 1 group and service the level 2 group, in STATA 16 [20]. A null model without explanatory variables was computed with mental health difficulties at baseline as the criterion variable, and the intraclass correlation coefficient (ICC) was calculated. The ICC was 32.33% (95% Confidence Interval or CI = 15.96–48.71%) indicating that there was significant service-level variation and confirming that multilevel regression was the correct analytical approach. To examine the associations with individual-level characteristics, two models were tested. In Model 1, demographic characteristics were entered as level-1 explanatory variables: economic disadvantage (with the least deprived quintile coded as the reference category to facilitate interpretation), grand-mean centred age, female gender, and ethnicity (with White British as the reference category as it was the largest group). In Model 2, clinical characteristics were added as level-1 explanatory variables: referral source (with primary care as the reference category as it was the largest group) and the 26 presenting difficulty variables (to avoid including underpowered variables, four variables were not included as they had a frequency of < 5%: selective mutism, toilet problems, developmental difficulties, and gender identity difficulties). The likelihood ratio test was used to compare successive models, and both were significantly better fits to the preceding model; Model 1: χ^2^(12) = 641.136, *p* < 0.001 and Model 2: χ^2^(35) = 860.38, *p <* 0.001.

To investigate predictors of treatment outcome (research question 2), two-level multilevel regressions were performed, with child as the level 1 group and service the level 2, with mental health difficulties at follow up as the criterion variable. In the null model, the ICC was 25.80% (CI = 8.70–42.90%). To determine treatment outcome, or mental health difficulties at follow up controlling for mental health difficulties at baseline, mental health difficulties at baseline was added as a level-1 explanatory variable in Model 1. The *z*-scores for mental health difficulties as baseline, and mean *z*-score, were computed again as this was a sub-sample of the overall sample. In Model 2, demographic characteristics were entered as level-1 explanatory variables: economic disadvantage, grand-mean centred age, female gender, and ethnicity. In Model 3, clinical characteristics were entered as level-1 explanatory variables: referral source, the 26 presenting difficulty variables, and grand mean centred number of care contacts. The likelihood ratio test was used to compare successive models, and all were significantly better fits to the preceding model; Model 1: χ^2^(1) = 641.13994.16, *p* < 0.001, Model 2: χ^2^(12) = 22.11, *p =* 0.0364, and Model 3: χ^2^(35) = 82.94, *p* < 0.001. It should be noted that using standardized criterion variables for both sets of analyses resulted in small coefficient estimates.

## Results

### Research question 1: what accounts for variation in baseline mental health difficulties?

Compared to children and adolescents from the least economically disadvantaged areas, children and adolescents from high (*β* = 0.09, 95% CI = 0.03–0.15) and the most (*β* = 0.10, 95% CI = 0.04–0.16) economically disadvantaged areas had higher levels of mental health difficulties at baseline. Compared to boys, girls had higher levels of mental health difficulties at baseline (*β* = 0.28, 95% CI = 0.24–0.32). Compared to younger children and adolescents, older children and adolescents had slightly higher levels of mental health difficulties at baseline (*β* = 0.02, 95% CI = 0.01–0.03). In terms of ethnicity, compared to White British young people, young people from mixed-race ethnic backgrounds had lower levels of mental health difficulties at baseline (*β* = − 0.15, 95% CI = – 0.26-–0.05). In terms of referral source, compared to young people referred through primary care, young people referred through social care/ youth justice (*β* = − 0.30, 95% CI = − 0.41-–0.20), mental health services (*β* = − 0.14, 95% CI = − 0.23-–0.05), or not known referral sources (*β* = − 0.09, 95% CI = − 0.17-0.00) had lower levels of mental health difficulties at baseline, although the CI for not known referral included 0 and therefore this finding should particularly be interpreted with caution. In terms of presenting difficulties, young people with social anxiety, generalized anxiety, panic disorder, agoraphobia, low mood, self-harm, or features of post-traumatic stress disorder, had higher levels of mental health difficulties at baseline than young people without these presenting difficulties (please see Table 2 for coefficients and CIs for presenting difficulties). In contrast, young people with specific phobia, conduct disorder, or risk management difficulties had lower levels of mental health difficulties at baseline than young people without these presenting difficulties.

**Table 2:** Multilevel regressions with demographic and clinical characteristics predicting baseline difficulties

	Beta	SE	*p*-value	95% CI	
**Economic disadvantage**					
Low vs. least	0.03	0.03	0.29000	−0.03	0.10
Lower vs. least	0.03	0.03	0.38700	−0.04	0.09
High vs. least	**0.09**	0.03	0.00600	0.03	0.15
Most vs. least	**0.10**	0.03	< 0.01	0.04	0.16
**Demographics**					
Female vs. male	**0.28**	0.02	< 0.001	0.24	0.32
Age	**0.02**	0.00	< 0.001	0.01	0.03
**Ethnicity**					
Another White background vs. White British	− 0.03	0.06	0.667	− 0.15	0.10
Any other ethnic group vs. White British	0.08	0.05	0.103	−0.02	0.19
Asian vs. White British	− 0.07	0.07	0.345	−0.20	0.07
Black vs. White British	−0.09	0.08	0.232	−0.24	0.06
Mixed-race vs. White British	**−0.15**	0.05	0.004	−0.26	−0.05
Not reported vs. White British	0.03	0.03	0.341	−0.03	0.08
**Referral source**					
Self-referral vs. pri. Care	−0.02	0.05	0.59100	−0.11	0.06
Education vs. pri. Care	−0.06	0.04	0.12500	−0.13	0.02
Social care/ youth justice vs. pri. Care	**−0.30**	0.05	< 0.001	−0.41	−0.20
Child health vs. primary care	−0.02	0.05	0.66600	−0.12	0.07
A&E vs. primary care	−0.07	0.04	0.07000	−0.15	0.01
Mental health vs. pri. Care	**−0.14**	0.05	0.00200	−0.23	− 0.05
Other vs. primary care	−0.06	0.03	0.08000	−0.13	0.01
Not known vs. primary care	**−0.09**	0.04	0.04100	−0.17	0.00
**Presenting difficulties**					
Social anxiety	**0.13**	0.02	< 0.001	0.08	0.17
Separation anxiety	0.02	0.02	0.27800	−0.02	0.07
Generalised anxiety	**0.13**	0.02	< 0.001	0.09	0.18
OCD	0.00	0.03	0.97000	−0.05	0.05
Panic	**0.16**	0.02	< 0.001	0.12	0.21
Agoraphobia	**0.07**	0.02	0.00400	0.02	0.12
Phobia	**−0.07**	0.03	0.01800	−0.12	− 0.01
Low mood	**0.24**	0.02	< 0.001	0.20	0.29
Repetitive behaviour	−0.03	0.03	0.34100	−0.09	0.03
Self-harm	**0.16**	0.02	< 0.001	0.11	0.20
Bipolar disorder	0.04	0.03	0.14000	−0.01	0.10
Psychosis	0.03	0.04	0.40100	−0.04	0.11
Substance use	0.05	0.04	0.14500	−0.02	0.12
ADHD	−0.02	0.02	0.31200	−0.07	0.02
Conduct disorder	**−0.09**	0.03	0.00100	−0.14	− 0.04
Risk	**−0.07**	0.03	0.02700	−0.13	−0.01
PTSD	**0.12**	0.02	< 0.001	0.07	0.17
Eating disorder	0.05	0.03	0.06900	0.00	0.10
Attachment	0.00	0.02	0.97400	−0.05	0.05
Peer relationships	0.03	0.02	0.12400	−0.01	0.07
Family relationships	−0.02	0.02	0.28900	−0.07	0.02
Health adjustment	−0.06	0.04	0.14500	−0.13	0.02
Self-care	0.01	0.03	0.80000	−0.06	0.07
Unexplained physical problems	0.06	0.04	0.18300	−0.03	0.14
Emerging personality disorder	0.01	0.03	0.64900	−0.04	0.07
Carer management	−0.02	0.02	0.50100	−0.06	0.03

*Note. N =* 5907 episodes of care from 14 services with 65–1667 episodes per service

Coefficients in bold are significant at least at the *p* < .05 level

*SE* Standard error, *CI* Confidence interval, *A&E* Accident and Emergency, *OCD* Obsessive compulsive disorder, *ADHD* Attention-deficit-hyperactivity-disorder, *PTSD* Post-traumatic stress disorder

### Research question 2: what are the predictors of treatment outcome?

Young people with higher levels of mental health difficulties at baseline also had higher levels of deterioration in mental health difficulties at follow up (*β* = 0.72, 95% CI = 0.67–0.76). After controlling for levels of mental health difficulties at baseline, compared to boys, girls had higher levels of deterioration in mental health difficulties at follow up (*β* = 0.09, 95% CI = 0.03–0.16). In terms of referral source, compared to young people referred through primary care, young people referred through social care/ youth justice (*β* = − 0.20, 95% CI = − 0.37-–0.03) had higher levels of improvement in mental health difficulties at follow up. Young people with social anxiety, panic disorder, low mood, self-harm, or family relationship difficulties had higher levels of deterioration in mental health difficulties at follow up than young people without these presenting problems (please see Table 3 for coefficients and CIs for presenting difficulties). Finally, young people with a greater number of care contacts had slightly higher levels of deterioration in mental health difficulties at follow up compared to young people with a lesser number of care contacts (*β* = 0.00195, 95% CI = 0.0.00074–0.00316). It should be noted that the coefficient was very small, meaning this finding should be particularly interpreted with caution.

**Table 3:** Multilevel regressions with demographic and clinical characteristics predicting difficulties at follow up

	Beta	Standard error	*p*-value	95% CI	
**Baseline mental health difficulties**					
Baseline mental health difficulties	**0.72**	0.02	< 0.001	0.67	0.76
Economic disadvantage					
Low vs. least	−0.04	0.05	0.51	−0.14	0.07
Lower vs. least	0.01	0.05	0.89	− 0.10	0.11
High vs. Least	0.00	0.05	0.92	− 0.10	0.09
Most vs. least	0.04	0.05	0.45	−0.06	0.13
**Demographics**					
Female vs. male	**0.09**	0.03	< 0.01	0.03	0.16
Age	0.01	0.01	0.38	−0.01	0.02
**Ethnicity**					
Another White background vs. White British	−0.06	0.09	0.52	−0.22	0.11
Any other ethnic group vs. White British	−0.09	0.08	0.28	−0.24	0.07
Asian vs. White British	−0.02	0.09	0.81	−0.20	0.16
Black vs. White British	−0.08	0.11	0.43	−0.29	0.12
Mixed-race vs. White British	−0.06	0.09	0.47	−0.23	0.11
Not reported vs. White British	−0.01	0.04	0.88	−0.09	0.07
**Referral source**					
Self-referral vs. pri. Care	−0.12	0.07	0.08	−0.25	0.01
Education vs. pri. Care	−0.01	0.06	0.83	−0.12	0.10
Social care/ youth justice vs. pri. Care	**−0.20**	0.09	0.02	−0.37	−0.03
Child health vs. primary care	0.06	0.08	0.44	−0.09	0.21
A&E vs. primary care	−0.07	0.06	0.23	−0.20	0.05
Mental health vs. pri. Care	−0.01	0.09	0.91	−0.18	0.16
Other vs. primary care	0.02	0.06	0.76	−0.09	0.13
Not known vs. primary care	−0.05	0.07	0.48	−0.18	0.09
**Presenting difficulties**					
Social anxiety	**0.08**	0.04	0.04	0.00	0.15
Separation anxiety	0.03	0.04	0.34	−0.04	0.11
Generalised anxiety	−0.03	0.04	0.34	−0.11	0.04
OCD	0.02	0.04	0.63	−0.06	0.10
Panic	**0.08**	0.04	0.04	0.00	0.15
Agoraphobia	−0.05	0.04	0.23	−0.12	0.03
Phobia	0.03	0.04	0.46	−0.05	0.12
Low mood	**0.08**	0.04	0.03	0.01	0.15
Repetitive behaviour	−0.02	0.05	0.71	−0.11	0.07
Self-harm	**0.07**	0.03	0.03	0.01	0.14
Bipolar disorder	0.00	0.05	0.98	−0.09	0.09
Psychosis	0.11	0.06	0.06	0.00	0.22
Substance use	−0.04	0.06	0.53	−0.16	0.08
ADHD	0.02	0.04	0.65	−0.06	0.10
Conduct disorder	0.01	0.04	0.73	−0.07	0.10
Risk	0.08	0.05	0.13	−0.02	0.18
PTSD	0.00	0.04	0.95	−0.08	0.08
Eating disorder	0.02	0.04	0.60	−0.06	0.11
Attachment	0.00	0.04	0.93	−0.07	0.08
Peer relationships	−0.02	0.03	0.61	−0.08	0.05
Family relationships	**0.08**	0.03	0.01	0.02	0.15
Health adjustment	−0.02	0.07	0.73	−0.16	0.11
Self-care	−0.07	0.06	0.26	−0.18	0.05
Unexplained physical problems	0.06	0.07	0.38	−0.07	0.19
Emerging personality disorder	0.00	0.04	0.93	−0.08	0.09
Carer management	0.01	0.04	0.83	−0.07	0.08
**Number of care contacts**					
Number of care contacts	**0.00195**	0.00062	< 0.01	0.00074	0.00316

*Note. N =* 1805 episodes of care from 10 services with 53–485 episodes per service

Coefficients in bold are significant at least at the *p* < .05 level

*A&E* Accident and Emergency, *OCD* Obsessive compulsive disorder, *ADHD* Attention-deficit-hyperactivity-disorder, *PTSD* Post-traumatic stress disorder

## Discussion

The aims of the present study were to examine characteristics that account for variation in levels of mental health difficulties at baseline and then predictors of treatment outcome. We conducted a secondary analysis of a large administrative dataset from child and adolescent mental health services in England.

Young people with higher levels of mental health difficulties at baseline also had higher levels of mental health difficulties at follow up. In terms of key characteristics that both accounted for variation in levels of mental health difficulties at baseline and were predictors of treatment outcome, girls had higher levels of mental health difficulties at baseline and of deterioration in mental health difficulties at follow up than boys. Compared to young people referred through primary care, young people referred through social care/ youth justice had lower levels of mental health difficulties at baseline and had higher levels of improvement in mental health difficulties at follow up. In terms of presenting problems, young people with social anxiety, panic disorder, low mood, or self-harm had higher levels of mental health difficulties at baseline, and higher levels of deterioration in mental health difficulties at follow up, compared to young people without these presenting problems. Although we found no evidence of association with levels of mental health difficulties at follow up, we did find that children and young people from areas with high, and the highest, levels of economic disadvantage had higher levels of mental health difficulties at baseline than children and young people from areas with the lowest levels of economic disadvantage. These findings may suggest that there is a need for children and young people from areas of higher levels of economic disadvantage to have earlier receipt of specialist mental health services. Currently, children and young people from such areas are receiving support when their difficulties have escalated to a higher level than children and young people from areas of lower levels of economic disadvantage.

The findings of the present research are consistent with previous research showing that higher levels of mental health difficulties at baseline are associated with higher levels of difficulties at follow up [14]. These findings also build on the troubling pattern in the literature that young women in particular are experiencing increased levels of mental health difficulties [12]. We found that social anxiety, panic disorder, low mood, and self-harm were associated with higher levels of mental health difficulties at baseline and at follow up. This is consistent with previous studies on common characteristics associated with lower treatment outcome and those that indicate high levels of clinical complexity [7, 13–15].

There is ongoing debate about how treatment outcome should be conceptualized and assessed [10, 11]. It is especially important to review how treatment outcomes are framed and measured with young people and particularly with those from minoritized groups who may be less likely to be represented in the evidence on which current treatment outcome approaches were developed. The findings of the present research did not show ethnic differences in treatment outcome, however such differences may have been masked due to the lack of data on structural inequalities in administrative datasets [9], especially as evidence shows structural inequalities in relation to accessing child and adolescent mental health services [5]. Future research should work with young people from minoritized ethnic groups and relevant community organizations so that administrative data can include information on inequalities that are meaningful to the experiences of individuals from minoritized ethnic groups.

Future research should examine the lack of significant association between economic disadvantage and mental health difficulties at follow up from the present research. This may possibly be accounted for by young people with the highest levels of economic disadvantage being more likely to have unmet needs and to be not known by child and adolescent mental health services, meaning they are not represented in administrative data. In the present research, young people from areas of higher economic disadvantage had higher levels of mental health difficulties at baseline compared to young people from areas of lower economic disadvantage, suggesting economic inequalities in receipt of specialist mental health support. Moreover, the findings of the present research suggest that young people referred through social care/ youth justice had lower levels of mental health difficulties at baseline and at follow up compared to young people referred through primary care. We are not able to explain why such differences were found in the present research. Future qualitative studies should examine if the types of outcomes measured in mental health services capture what young people and professionals think are important outcomes and reflect the reasons for which young people receive mental health services through these pathways.

Limitations of the present research include the relatively small sample sizes, meaning that the findings may not reflect all young people accessing child and adolescent mental health services. Although we restricted the analysis to only measures of depression and anxiety for conceptual and operationalisation consistency, future research is needed to examine the factor structure of the five subscales used to determine the extent to which items load onto the same factor. Similarly, using a subsample to examine mental health difficulties at follow up means the groups in the baseline and follow up analyses are not entirely comparable. More general limitations of using administrative data are also relevant to the present research [21]. Moreover, the use of complete case analysis to manage missing data, especially on mental health difficulties at follow up, may mean there are systematic differences in those with and without these data. Future research examining such patterns and differences is encouraged, working towards consistency in how missing data are handled in administrative child mental health records. We assessed presenting difficulties using two different types of clinician reports, Current View questionnaire [8] presenting problems and ICD-10 free-text diagnoses mapped on to the Current View presenting problems. Inaccuracies in ICD-10 recording and inconsistencies in mapping across the two sources are other potential limitations. Nevertheless, this approach resulted in a more comprehensive assessment of presenting difficulties than would have been possible with one source alone. Future research should examine different types of care provided, which was not available in the present dataset, to examine whether predictors of treatment outcome differ across treatment modalities.

Notwithstanding the above limitations, the present research identified predictors of treatment outcome in a large and recent administrative dataset from child and adolescent mental health services in England. Based on the findings presented in this paper, services seeing higher proportions of young people with higher levels of mental health difficulties at baseline, social anxiety, panic disorder, low mood, or self-harm may be expected to show lower levels of improvement in mental health difficulties at follow up.

## Data Availability

Requests to access the data from which the data for this paper were derived can be made to NHS Digital through the Data Access Request Service.

## Abbreviations

GAD-7: Generalised Anxiety Disorder 7
ICC: Intraclass Correlation Coefficient
IDACI: Income Deprivation Affecting Children Index
PHQ-9: Patient Health Questionnaire 9
RCADS: Revised Children’s Anxiety and Depression Scales
SDQ: Strengths and Difficulties Questionnaire
